# Cascade aza-Michael Addition-Cyclizations; Toward Renewable and Multifunctional Carboxylic Acids for Melt-Polycondensation

**DOI:** 10.3389/fchem.2019.00729

**Published:** 2019-11-14

**Authors:** Geert J. Noordzij, Carolus H. R. M. Wilsens

**Affiliations:** ^1^Chemelot InSciTe, Geleen, Netherlands; ^2^Faculty of Science and Engineering, Aachen-Maastricht Institute of Biobased Materials (AMIBM), Maastricht University, Geleen, Netherlands

**Keywords:** aza-Michael addition-cyclization, *N*-alkyl-pyrrolidone methylcarboxylate, itaconic acid, aconitic acid, renewable monomers, polycondensation

## Abstract

Although the aza-Michael addition reaction on various unsaturated (di-)carboxylic acids and esters of, for example, itaconic acid, is well-known, the consecutive cyclization reaction has not received much attention in literature. The products of this aza-Michael cascade reaction, being mono- or di-carboxylic acid or ester functionalized *N*-alkyl-pyrrolidone structures, prove interesting for melt-polycondensation reactions as they exhibit excellent stability at elevated temperatures. In other words, this reaction is a toolbox for the generation of renewable monomers and, in turn, polymers with tunable physiological properties. Therefore, this work provides an overview of the state-of-the-art of the cascade aza-Michael addition-cyclization reactions on biobased unsaturated acids and esters, and their use in polymerization reactions. Furthermore, we extend this overview with the cascade aza-Michael addition-cyclization reaction of *trans*-trimethyl aconitate with di-amines to form a tetra-functional *N*-alkyl-*bis*-(pyrrolidone dimethylcarboxylate), which exhibits excellent thermal stability and could effectively be used as monomer in polycondensation reactions. Importantly, the aza-Michael addition reaction between primary amines and *trans*-trimethyl aconitate can be considered a click-reaction; it proceeds quantitatively within minutes under ambient conditions and follows the principles of green chemistry.

## Introduction

**Scheme 1:**
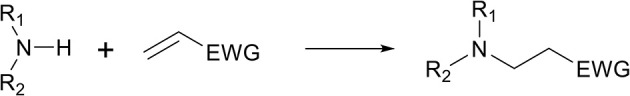
Generalized reaction scheme of the aza-Michael addition reaction between a primary (R_1_ = H, R_2_ ≠ H), or a secondary amine (R_1_, R_2_ ≠ H), and an electron deficient alkene with an electron withdrawing group (EWG).

Recently, polymeric materials are increasingly (re-)evaluated in academia, industry, and society as they are reportedly causing pollution and negatively impact the environment (Mülhaupt, 2013). One route to improve the sustainability and environmental impact is to consider the principles of green chemistry, both for the development of monomers and for the development of polymer materials. Example of components that need to be considered are the atom efficiency, use of renewable feedstock, design to recycle, use of less-hazardous chemical synthesis, solvents, and catalysts (Anastas and Kirchhoff, 2002). Particularly interesting transformations that can be considered green reactions are click-reactions, as is coined by Sharpless and coworkers in 2001 (Kolb et al., 2001). One of such click-reactions in organic chemistry is the well-known aza-Michael reaction (Scheme 1), which is an addition reaction involving a nucleophilic amine (Michael donor), and an electron deficient alkene (Michael acceptor). This reaction is widely employed in the synthesis of biologically active compounds and, when using the right reactants, readily proceeds at room temperature (Rulev, 2011).

Not surprisingly, the aza-Michael addition has been used in both monomer and polymer synthesis, and has been reviewed in three recent publications regarding the use of the aza-Michael reaction applied to the general polymer domain (Mather et al., 2006), to the general chemistry domain (Rulev, 2011), and to silicon-based polymers (Genest et al., 2017). The aza-Michael addition reaction is particularly interesting for modification of renewable monomers, as numerous renewable molecules contain primary or secondary amines and/or unsaturated groups. Furthermore, the selectivity of the aza-Michael addition reaction can be used to convert multi-functional molecules into new biobased monomers for polymerization reactions. One example of such a monomer is itaconic acid or its methyl ester, dimethyl itaconate, which can function as a Michael acceptor. This molecule is prone to participate in the aza-Michael addition reaction, and subsequently undergoes a cascade cyclization reaction to form a highly stable *N*-alkyl-pyrrolidone carboxylic acid (Paytash et al., 1950). In other words, this reaction allows for the transformation of an amine group into a rigid and thermally stable carboxylic acid or methyl ester functionalized monomer, which can be used for further polymerization reactions.

The previously described cascade cyclization reaction to form new stable monomeric intermediates via the aza-Michael reaction has not been described in previous reviews. This is unexpected, as, besides itaconic acid, several other monomers such as fumaric acid, maleic acid, *trans*-β-hydromuconic, and aconitic acid, prove susceptible to participate in the aza-Michael (cascade) reaction to yield highly promising monomers for further polymerization. Therefore, in this work we provide an overview of the current research activities on renewable unsaturated acids and esters for monomer synthesis via both the aza-Michael addition and the cascade cyclization reaction, and elaborate on their use in melt-polycondensation reactions. Furthermore, we extend the state-of-the-art with new findings, highlighting the potential of the aza-Michael cascade reaction for *trans*-trimethyl aconitate and demonstrate its potential in polymerization reactions.

## Essentials for aza-Michael Cascade Reaction

### Michael Donors

The reactivity of different amines as Michael donor has been studied for several Michael acceptors, such as acrylates (Wu et al., 2004), methyl acrylates (Zou and Jiang, 2008), and vinyl phosphonates (Matveeva et al., 2008). Several factors influence the reactivity of the amine as Michael donor, such as nucleophilicity, steric hindrance, and aromaticity. In general, cyclic secondary amines such as piperidine are stronger nucleophiles than primary amines due to the inductive effect of the *N*-substituted alkyl groups, and can therefore react faster as Michael donor. However, bulky secondary amines such as dibutylamine are slower to react than primary amines due to steric hindrance. In turn aromatic amines such as aniline showed no reactivity as Michael donor at room temperature without catalyst, and therefore would require harsher conditions to react (Ai et al., 2010). A comprehensive overview of the different reactivity of amines as Michael donor has been extensively discussed in an excellent review paper by Ganachaud and coworkers and we refer to this work for more information on this topic (Genest et al., 2017).

### Michael Acceptors

**Scheme 2:**
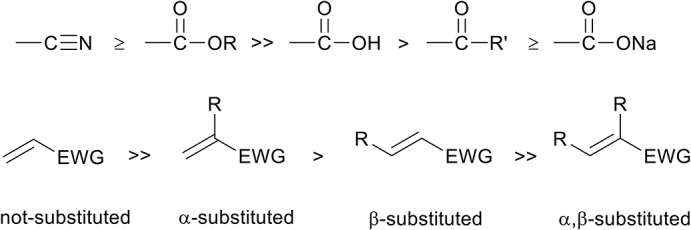
Top, from left to right: the strongest electron withdrawing group to less strong electron withdrawing group. The stronger the electron withdrawing group on the β-position of the unsaturated bond, the higher the reactivity toward the aza-Michael addition reaction. Bottom, reactivity dependency of the Michael acceptor as a function of the degree of α,β-substitution.

The capability of unsaturated monomers to act as Michael acceptor depends on the nature of this unsaturated bond. Only electron deficient unsaturated bonds are capable of accepting an electron from the nucleophilic Michael donor, and the degree of electron deficiency is largely determined by electron withdrawing groups (EWG) on the β-position relative to the unsaturation. It was shown that nitrile groups and methyl esters have the highest electron withdrawing effect (Scheme 2, top) (Critchfield et al., 1956), and therefore unsaturated monomers with these EWGs in the β-position are highly susceptible toward to aza-Michael addition reaction. This can be seen by the high amount of publications of the aza-Michael addition on unsaturated monomers such as acrylates and acrylonitriles (Genest et al., 2017).

The electron deficiency of the unsaturation is also dependent of the degree of alkyl-substitution on the monomer. Alkyl substitutions, which have an electron donating effect, on both the α-, as β-position can slow down the aza-Michael addition reaction (Scheme 2, bottom) (Genest et al., 2017). Generally it is reported that with a higher degree of alkyl-substitution, an increase in reaction temperature is required for the aza-Michael addition to occur (Critchfield et al., 1956).

### Cascade Cyclization Reaction

**Scheme 3:**
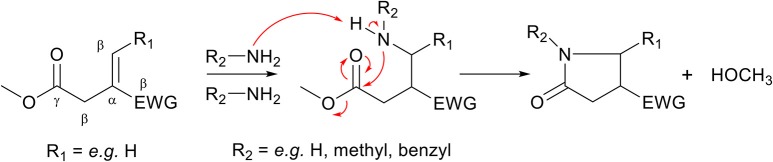
Schematic overview of the cascade reaction of the aza-Michael addition of ammonia or a primary amine to an unsaturated monomer with an ester in the γ-position **(left)**. The aza-Michael addition reaction is followed by an autocatalyzed (by primary amines) amidation-cyclization step **(middle)** which, with the loss of a condensate molecule, forms a 5-membered *N*-substituted pyrrolidone ring **(right)**.

Apart from the aza-Michael addition reaction, monomers with an ester group on the γ-position relative to the unsaturated bond can undergo a cascade reaction when ammonia or a primary amine is used in the aza-Michael addition reaction. The formed secondary amine in the aza-Michael adduct (Scheme 3, middle) can act as a nucleophile, and undergoes an autocatalyzed intramolecular amidation-cyclization reaction with an ester spaced four carbons away (γ-position) to form a highly stable 5-membered *N*-substituted pyrrolidone ring (Scheme 3, right). This cascade reaction is well-known to occur with the biobased monomer dimethyl itaconate (Scheme 3, EWG = methyl-ester, R_1_ = H), and readily reaches full conversion when conducted at elevated temperatures, as will be explained in more detail later in this work. The cyclization reaction is irreversible under normal synthesis conditions, and thereby avoids any potential retro aza-Michael reactions which have been observed in other systems (Baruah et al., 2016). Although the cyclization step does include the loss of a condensation molecule, the reaction can proceed fast, highly selective and with high yields, both in bulk or in green solvents such as methanol or water, hence following the principles of green chemistry.

## Renewable Monomer Selection

Renewable monomers with an unsaturation are widely reported and examples include biobased acrylates, terpenes, furfural/furfuryl alcohol based monomers, glycerides and related unsaturated fatty acids, and unsaturated esters. As described in the previous section, the electron deficiency of the double bond is crucial in order for these molecules to be a candidate for the aza-Michael addition reaction. Monomers without EWG in close vicinity to the unsaturation are typically unreactive toward the aza-Michael addition, as is the case for unsaturated fatty acids. Some lignin-derived and terpene-derived unsaturated monomers do have vicinal EWGs but usually lack additional functional groups apart from an unsaturated bonds, thereby often limiting their use to radical polymerizations (Wilbon et al., 2013; Satoh, 2015). Conjugated or aromatic unsaturated double bonds such as in muconic acid, 2,5-furandicarboxylic acid, 2,5-hydroxylmethylfurfural, furfuryl, and furfurylalcohol are greatly stabilized by conjugation, and thereby have no reported reactivity toward the aza-Michael addition reaction. Furthermore, 2,5-hydroxymethylfurufral, furfurylalcohol, and some of their derivatives have functional groups (e.g., aldehydes) which react faster with amines, thereby competing with, or even overruling the aza-Michael addition reaction.

One class of biobased unsaturated monomers which are (highly) susceptible toward aza-Michael addition reactions are unsaturated (di-)esters. Importantly, unsaturated (di-)esters can retain multi-functionality after the aza-Michael addition with mono- or di-amines, and can therefore subsequently be used in the preparation of linear, branched, and cross-linked polymers. Since to date this part has not received much attention in academic literature, in the next section we provide an overview on the aza-Michael addition reaction involving unsaturated (di-)esters from biomass, with a special attention for the potential use in the synthesis of (biobased) polymers. Note, several relevant unsaturated esters from biomass have been excluded from this overview, the reason being that (potentially) biobased acrylates, obtainable via glycerol (Della Pina et al., 2011), have already been included in a review regarding the aza-Michael additions on acrylate monomers (Genest et al., 2017).

**Scheme 4:**
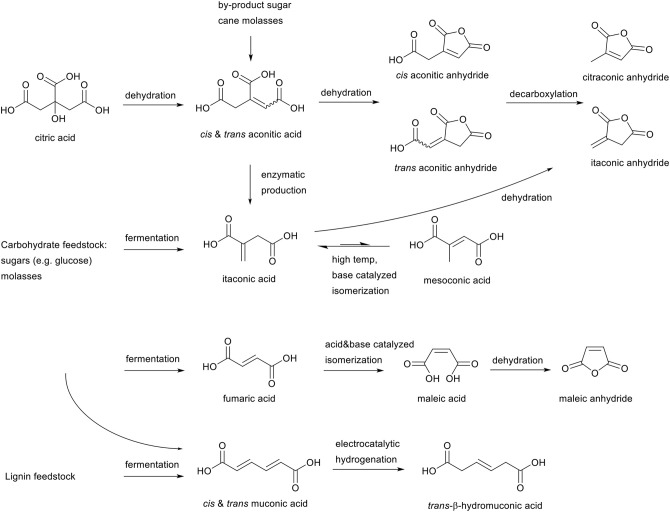
Overview scope of literature research of unsaturated esters from biomass for the aza-Michael addition reaction.

The aforementioned selection criteria on reactivity leaves several candidates which are proven to be susceptible, or expected to be susceptible, toward the aza-Michael addition reaction under mild reaction conditions (Scheme 4). Firstly, aconitic acid (*cis* and *trans*) is a tri-functional carboxylic acid with an unsaturation in the backbone, and can be obtained as a by-product form the sugar-cane industry (Kanitkar et al., 2013), or after dehydration of biobased citric acid. Further dehydration of aconitic acid leads to the corresponding *cis* and *trans* aconitic anhydrides, with the unsaturation retained within the anhydride ring, or in the side chain. Thermal treatment of these anhydrides can lead to decarboxylation to form citraconic anhydride from *cis*-aconitic anhydride, or itaconic anhydride from *trans*-aconitic anhydride.

Itaconic acid, an unsaturated ester with the unsaturation *exo* on the backbone can readily be obtained from fermentation of several carbohydrate feedstocks such as glucose, but also from aconitic acid. Itaconic acid is produced on an industrial scale, and is for example used in superabsorbent polymers by Itaconix®. Itaconic acid can be base catalyzed at elevated temperatures to isomerize toward mesaconic acid, and dehydration of itaconic acid can lead to itaconic anhydride as shown before in the aconitic acid cycle.

Other relevant biobased unsaturated esters are fumaric and maleic acid, which can be obtained by fermentation of carbohydrate biomass feedstocks (Mariscal et al., 2016). Maleic acid is the structural isomer of fumaric acid and can be obtained via both acid and base catalyzed isomerization. Furthermore, maleic acid can be dehydrated to form maleic anhydride.

Lastly, muconic acid, a C6 acid with a double unsaturation in the main chain, can be obtained from fermentation of both carbohydrate feedstocks as from lignin feedstocks (Vardon et al., 2016). Electrochemical hydrogenation of muconic acid leads to *trans*-β-hydromuconic acid, with one unsaturation left in the main chain. Though currently commercially not yet relevant, in recent years it has gained scientific interest as muconic acid provides a potential route for the production of biobased terephthalic acid (Lu et al., 2016), and *trans*-β-hydromuconic acid for adipic acid (Matthiesen et al., 2016a,b).

## State of the Art

### aza-Michael Addition on Itaconic Acid

**Scheme 5:**
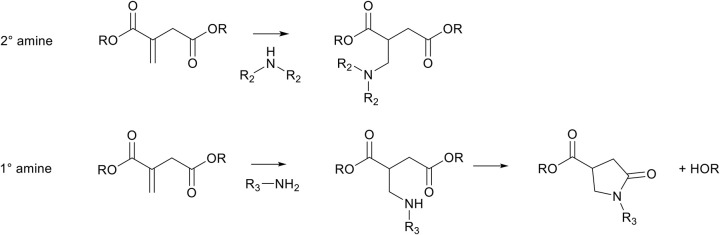
Schematic overview of possible aza-Michael addition reactions on itaconates. Top, a secondary amine (e.g., diethylamine, piperidine) can add to itaconates to form the aza-Michael adduct. Bottom, primary amines (e.g., ethylamine, ethanolamine) can add to itaconates to form the aza-Michael adduct which subsequently ring-closes to form *N*-functionalized mono-pyrrolidone.

Itaconic acid and dimethyl itaconate are highly susceptible toward the aza-Michael addition reaction, and, when reacted with secondary amines, form a good platform to modify the backbone whilst retaining the dimethylcarboxylate functionality. For example, the addition of diethylamine to itaconate is highly selective toward the β-position, and therefore adds only to the *exo*-position (Farmer et al., 2018a). This modification can be performed both prior to polymerization of the itaconates (Pellis et al., 2019), or after polymerization (Scheme 5, top) (Chanda and Ramakrishnan, 2015; Farmer et al., 2018b). Furthermore, the aza-Michael addition reaction is reported to be irreversible at the employed reaction conditions (room temperature), and only showed minor reversibility at elevated temperatures (Pellis et al., 2019). The potential aza-Michael addition reaction with mesaconate does not occur at all, which shows that, despite the isomerization of itaconate to mesaconate, the aza-Michael reaction is specific to the β-position on itaconates. Nevertheless, Clark and coworkers reported that eventually all mesaconate isomerizes back to itaconate when the reaction was left stirring long enough, which then formed the aza-Michael adduct with almost full conversion (Farmer et al., 2018a).

The aza-Michael addition reaction between itaconic acid and aliphatic primary amines is well-known to subsequently undergo a cascade intramolecular cyclization step to form a *N*-substituted pyrrolidone ring (Scheme 5, bottom, R_3_ = alkyl). This has for example been used to make *N*-methyl-pyrrolidone using methylamine, or to make surfactants with amines with long aliphatic chains (Okada et al., 2009; Malferrari et al., 2015). Keeping eye on further use of such monomers in polycondensation reactions, this cascade reaction is rather interesting because it is an irreversible reaction, and the newly obtained pyrrolidone-ring is thermally stable and resistant to both high and low pH. Therefore, the stability of this newly formed group can be promising to protect otherwise labile amine and/or acid functionalities. For example, as is reported in previous work, the potentially renewable *cis*/*trans* 1,3-cyclopentanediamines are prone to degrade (by oxidation and dehydration) at elevated temperatures, similar to their diol counterparts *cis*/*trans* 1,3-cyclopentanediols (Noordzij et al., 2018). This instability makes the use of these monomers via melt-polycondensation reactions challenging as the final products are heavily colored and either have low molecular weights or are cross-linked (Noordzij et al., 2018). Instead, when reacting these monomers with itaconic acid, thermally stable dicarboxylic acid monomers are obtained that can readily be used in melt-polycondensation reactions (Noordzij et al., 2019). In addition, the presence of the pyrrolidone ring adds to the rigidity of the newly obtained monomers, leading to a change in physicochemical properties.

**Scheme 6:**
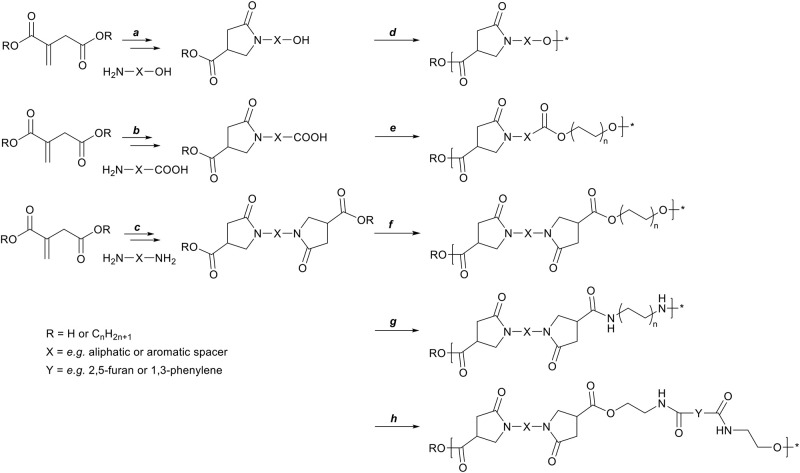
Schematic overview of preparing polymer precursors via the aza-Michael addition reaction on itaconates. First: itaconates with amino-alcohols (a) can generate a carboxyl-alcohol functional monomers which can be used in a self-polycondensation reaction (d). Second: itaconates with amino-acids (b, e.g., p-amino-benzoic acid, phenylalanine) can generate asymmetric dicarboxylic acid functional monomers which can be polymerized with a diol (e). Third: itaconates with diamines (c, e.g., ethylene diamine, *p*-phenylene diamine) can generate symmetrical dimethylcarboxylate functional monomers which can be polymerized with either diols (f), diamines (g), or bis-oxazolines (h) to yield polyesters, polyamides, or poly(ester amide)s, respectively.

In order for such *N*-functionalized pyrrolidone-methylcarboxylates to be of use in further polymerization reactions, di- or multi-functional amines are required to retain the multi-functionality. The synthesis of such multifunctional monomers from itaconates and various amines, and the subsequent use in polymer synthesis has been known for some time, but has seen a rise in publications in the recent years. Scheme 6 summarizes the published itaconate-based monomer synthesis and subsequent polymerizations thereof: The reaction between itaconates and an amino-alcohol (**a**, e.g., with ethanolamine) leads to a carboxyl-alcohol functional pyrrolidone, which can be polymerized by self-polycondensation (**d**) into an amorphous polyester with a glass transition temperature (*T*_g_) of 60°C (Qi et al., 2016). The reaction between itaconates and an amino-acid (**b**, e.g., with *p*-amino-benzoic acid or *L*-phenylalanine) leads to asymmetrical dicarboxylic acid functional pyrrolidone, which can be polymerized with a diol (**e**, e.g., with 1,3-propanediol) to yield amorphous polyesters with a *T*_g_ ranging between 96 and 48°C (**b** = *p*-aminobenzoic acid or *L*-phenylalanine, respectively) (Miller and Qi, 2019). The reaction between itaconates and aliphatic diamines (**c**, e.g., with 1,2-ethylene diamine or 1,12-dodecanediamine) leads to symmetrical bis-(pyrrolidone-methylcarboxylate) (BPDA) monomers. These BPDA monomers can be polymerized with various diols (**f**, e.g., with 1,3-propanediol or 1,12-dodecanediol) into amorphous polyesters with a *T*_g_ ranging from 62 to −22°C (Qi et al., 2016; Noordzij et al., 2019). BPDA monomers obtained from rigid diamines (**c**, e.g., with *p*-phenylenediamine or *trans*-cyclohexanediamine) can be polymerized into semi-crystalline polyesters with various diols (**f**, e.g., with 1,8-octanediol), with a *T*_g_ between 65 and 33°C, and a melting temperature (*T*_m_) between 214 and 149°C (Noordzij et al., 2019). Furthermore, the reaction between itaconates and aliphatic or aromatic diamines (**c**, e.g., with 1,6-hexanediamine or *p*-phenylenediamine) followed by subsequent polymerization with various aliphatic or aromatic diamines (**g**, e.g., with 1,6-hexanediamine or *p*-phenylenediamine), leads to the formation of high *T*_g_, amorphous polyamides. Such polyamides were either obtained via salt-formation between itaconic acid and diamines, with subsequent polycondensation to yield mono-pyrrolidone polyamides (Ali et al., 2013, 2014, 2019; Wang et al., 2014; He et al., 2016). Alternatively, the BPDA monomers were first isolated and subsequently polymerized via polycondensation with various amines into amorphous polyamides (Avny et al., 1972; Gozlan et al., 1988; Ayadi et al., 2013; Noordzij et al., 2019).

Lastly, BPDA monomer based on 1,10-dodecanediamine (**c**) was co-polymerized into poly(ester amide)s with sebacic acid, 1,10-dodecanediamine, and 1,4-butanediol, where it was shown that adding BPDA monomer could suppress crystallization (Wang et al., 2016). BPDA monomers based on aliphatic diamines (**c**, e.g., is 1,2-ethylene diamine or 1,12-dodecanediamine) can be polymerized with various bis(2-oxazoline)s (**h**, e.g., Y is 2,5-furan or 1,3-phenylene) to yield linear or cross-linked amorphous poly(ester amide)s with a *T*_g_ ranging from 60 to 120°C (Roy et al., 2018, 2019).

**Scheme 7:**
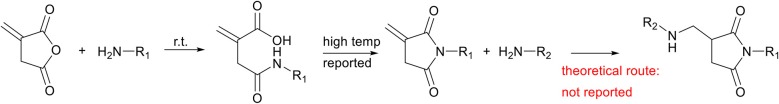
Schematic overview of the reaction of itaconic anhydride with a primary amine (left), which after thermal treatment yields itaconimides (middle right), with the unsaturation still present. Although several Michael addition reactions on this double bond have been described, to date no aza-Michael addition reaction has been described on itaconimides (right).

The last member of the itaconate derivatives is itaconic anhydride, which does not show reactivity toward the aza-Michael addition reaction because anhydrides are more susceptible to undergo a reaction with amines than the unsaturated bond. The ring-opening of the anhydride results in the formation of an amide and a free carboxylic acid group (Scheme 7, middle left), which in turn leads to the formation of itaconimides at elevated temperatures (Scheme 7, middle right). Interestingly, these itaconimides retain their unsaturated bond, and several Michael additions on this double bond have been described (Ahire and Mhaske, 2017). However, to date and to the best of our knowledge, no aza-Michael addition has been reported in literature on these itaconimides (Scheme 7, right).

### aza-Michael Addition on Polyitaconate

**Scheme 8:**
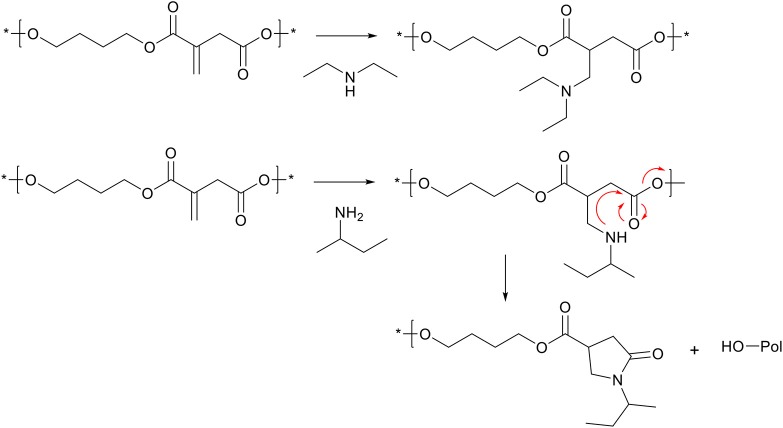
Schematic overview on aza-Michael addition on poly(butylene-itaconate). Top, Modification of *exo*-pendant unsaturation with a secondary amine. Bottom, modification of *exo*-pendant unsaturation with a primary amine, which is followed by the cyclization step leading to the pyrrolidone ring, and simultaneous chain-scission of the polymer backbone. Reaction arrows (red) are added for clarity.

Itaconic acid has been used plentiful in direct polycondensation with diols, to yield polyesters with the unsaturated bond *exo* on the main chain (Robert and Friebel, 2016). This unsaturation can undergo the same aza-Michael addition reactions as previously described, and therefore the (co-)polyester can be modified with secondary amines such as diethylamine (Scheme 8, top) (Lv et al., 2014; Chanda and Ramakrishnan, 2015; Hoffmann et al., 2015; Guarneri et al., 2019). However, when using primary amines, the cascade cyclization reaction can take place, with part of the polymer chain as the leaving group, effectively leading to chain scission (Scheme 8, bottom) (Farmer et al., 2018b). This chain scission reaction proved to be a useful tool for reactive degradation of itaconic acid based polyesters (Lv et al., 2014; Farmer et al., 2018b). Furthermore, the secondary amine formed during the aza-Michael addition is expected to be able to participate in second aza-Michael addition with another unsaturation in the polymer backbone, effectively generating cross-links. However, no references of this reaction are found in literature on itaconic acid based polymers, possibly the result of steric hindrance and likely favoring the cascade cyclization reaction.

### aza-Michael Addition on Fumaric Acid and Maleic Acid

**Scheme 9:**
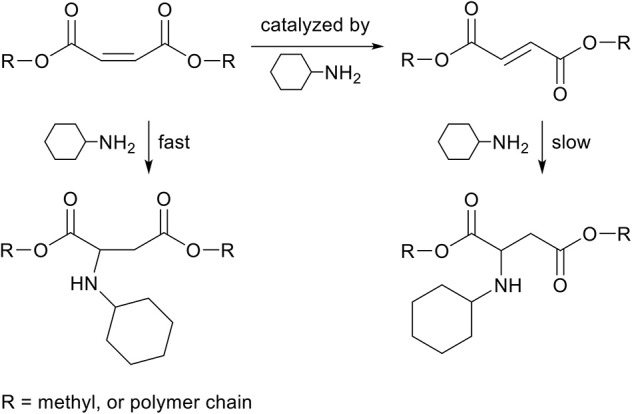
Schematic overview of the reactions between dimethyl maleate and cyclohexylamine. Both the aza-Michael addition (left) as the isomerization toward dimethyl fumarate (top) occur fast. The aza-Michael addition to dimethyl fumarate (right) occurs at a lower rate.

Fumaric acid and maleic acid are structural isomers, and it is well-known that fumaric acid can be acid-catalyzed toward maleic acid (Davies and Evans, 1956). However, similarly to itaconate and mesaconate, this isomerization can also be base-catalyzed by the same amine used for the aza-Michael addition reaction. Such isomerization can happen during polymerization, leading to a change in composition of monomers, and therefore to a change of the desired polymer properties (Jada, 1985). In contrary to mesaconate, both fumarate and maleate act as aza-Michael acceptor, though kinetic studies show that maleate reacts much faster than fumarate (Scheme 9), which is likely caused by the higher thermodynamic stability of fumarate (Bláha et al., 2018). The combined effect of the presence of two EWGs on the β-position in respect to the unsaturated bond, and thermodynamically less stable maleate isomer, ensures a high reactivity toward the aza-Michael addition reaction on maleates. The reaction can be performed solvent-free, catalyst-free, and at room temperature, showing the potential of this green transformation (Bosica and Debono, 2014). Some research has been reported on the reversibility of the aza-Michael addition reaction to maleates, and it was shown that the reversible aza-Michael addition only occurred after prolonged heating at 85°C when using secondary amines (Pellis et al., 2019). In other words, we have found no evidence in literature that the reverse aza-Michael addition proceeds when making use of primary amines and maleates. Furthermore, polymers with varying ratios of fumarate and maleate in the backbone were modified with various primary amines at room temperature, and solely the maleate proved reactive toward aza-Michael addition reaction under these conditions (Yu et al., 2018).

Modification of the maleate monomer with an amine, or diamine, could lead to interesting new multi-functional monomers which in turn can be used in various polymerization reactions (Tapaswi et al., 2014). However, there are only a very few publications reporting on this transformation for use in polymer synthesis. Similarly, modification of maleate and fumarate containing polymers to achieve desired polymer properties are very limited. Since it was shown that these reactions can be fast, selective, clean, and high yielding, there still seems to be a great unused potential for maleates and fumarates for the synthesis of biobased polymers with enhanced functionality.

Similar to itaconic anhydride, maleic anhydride shows no reactivity toward the aza-Michael addition reaction because the anhydride reacts much faster with amines. Interestingly though, unlike for itaconimides, the imide of maleate is reported to be susceptible toward aza-Michael addition reactions. By reacting two equivalents of maleic anhydride with a diamine structure, a bis-maleimide can be obtained, which can be subsequently polymerized with diamines via the aza-Michael addition reaction (Scheme S1, ESI) (Dolci et al., 2016). Even though commercially successful polymers (Kerimid®) (Fullerton et al., 1988) have been prepared this way, this aza-Michael addition reaction is mostly only selective for aromatic amines, and not for aliphatic amines (Gherasim and Zugrǎvescu, 1978). As a result, this potentially limits the clean and selective transformation since aza-Michael reactions with aromatic amines require harsh reaction conditions, and aromatic diamines are rarely renewable.

## Recent developments

### aza-Michael Addition on Aconitic Acid

**Scheme 10:**
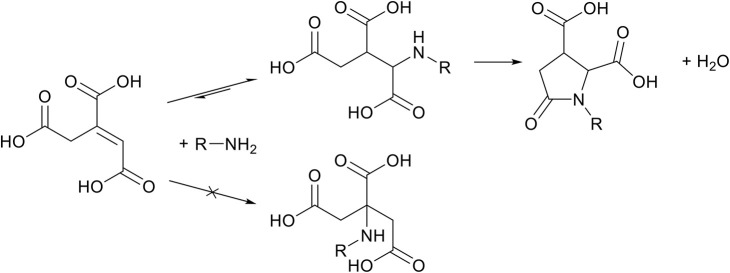
Schematic overview of the aza-Michael addition reaction between aconitic acid and an amine, followed by an autocatalyzed cascade amidation-cyclization step to form a *N*-substituted-pyrrolidone ring with 2 carboxylic acid groups on the ring.

Aconitic acid, which is mainly obtained in the *trans*-configuration from sugar cane molasses (Montoya et al., 2014), is a very interesting unsaturated ester from biomass due to the tri-carboxylic acid functionality. Both *cis*- and *trans*-isomers have seen some use as monomer in polycondensation reactions. For example, mainly *cis*-aconitic acid is used for its hydrolytic capacity in polyamides, leading to water-soluble polymers which could be hydrolyzed at low pH (DuBois Clochard et al., 2000; Zloh et al., 2003). In contrast, *trans*-aconitic acid is more stable as can be deduced by the lower hydrolysis rate (Dinand et al., 2002) and has successfully been used in the synthesis of hyperbranched polymers (Cao et al., 2011). Despite the better stability of *trans*-aconitic acid compared to *cis*-aconitic acid, thermal decarboxylation readily occurs from 105°C (Sepulchre and Sepulchre, 1997; Noordover et al., 2007) and higher, generating itaconic acid (Scheme 4), thereby limiting its use in high temperature applications. Considering the thermal instability, aconitic acid is an excellent candidate for the cascade aza-Michael addition-cyclization reaction to form the thermally stable pyrrolidone ring (Scheme 10). Interestingly this *N*-substituted-pyrrolidone obtained from aconitic acid has two carboxylic acid groups on the ring, instead of one as is obtained from itaconic acid.

**Scheme 11:**
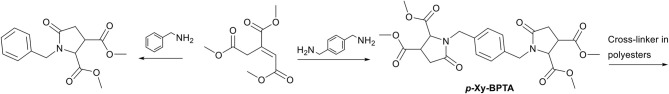
Schematic overview of model aza-Michael addition reaction on trans-trimethyl aconitate as presented in this work.

To the best of our knowledge the aza-Michael addition between an amine and *trans*-trimethyl aconitate, followed by the cascade cyclization step, has been reported only four times (Joentgen et al., 2002; Okada et al., 2009; Schäfer et al., 2009; Singh et al., 2016). All reported amines were primary mono-amines (e.g., benzylamine), to yield a *N*-substituted mono-pyrrolidone-dimethylcarboxylate, and found potential use as surfactant (Okada et al., 2009). The reported synthesis methods were in methanol as solvent, at low temperatures (ranging from ambient temperatures up to 40°C), and reported moderate to high (43–85%) isolated yields after column chromatography. Since the potential of aconitic acid to generate multi-functional carboxylic acids is high, we studied this reaction in more detail with benzylamine, and *para*-xylylenediamine as model compounds (Scheme 11). Details on the experimental methods and analysis are provided in the ESI.

**Figure 1:**
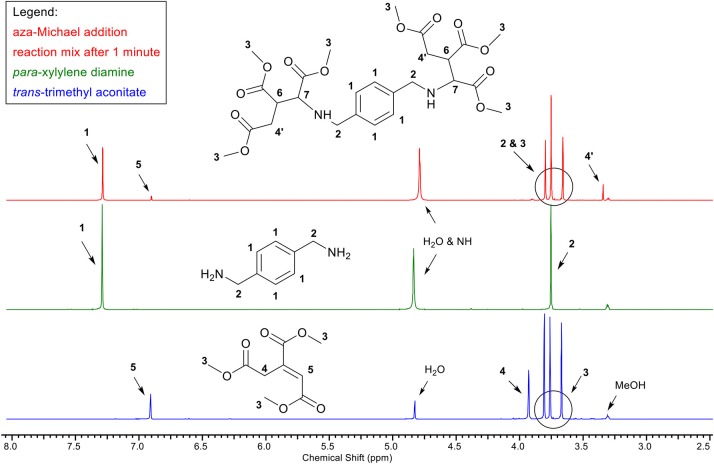
^1^H-NMR analysis depicting the aza-Michael addition between trans-trimethyl aconitate (1 equiv.) (blue, bottom) and para-xylylene diamine (0.95 equiv.) (green, middle). The reaction mixture (red, top), was measured after 1 min reaction time at room temperature in deuterated methanol as reaction medium and NMR solvent. The reduction of signal five indicates the completed aza-Michael addition reaction after 1 min reaction time.

Upon addition of benzylamine or *para*-xylylenediamine to a small excess of *trans*-trimethyl aconitate (Scheme 11) in methanol at room temperature, the colorless solution turned yellow immediately. Figure 1 bottom, shows the ^1^H-NMR spectrum of *trans*-trimethyl aconitate, with the protons of the double bond (**5**) at 6.9 ppm. ^1^H-NMR was immediately performed (Figure 1, top) after addition of a 0.95:1 molar equivalent of *para*-xylylenediamine (Figure 1, middle) and it was observed that resonance **5** has decreased according to the molar equivalent of diamine added. These findings indicate that the aza-Michael addition reaction already reaches full conversion immediately after mixing of the reactants. Note, the ^1^H-NMR-spectrum of the expected aza-Michael adduct (Figure 1, top) is missing signals for protons **6** and **7**, which likely underwent deuterium-exchange due to their slightly acidic nature, which is supported by the rise in the H_2_O signal. LC-MS studies indicated the presence of minor impurities, which, based on their mass, were identified as products where amide formation occurred between the amine and one of the methyl-esters of *trans*-trimethyl aconitate. This is considered an undesired side-reaction as it depletes the amines required for the aza-Michael addition. However, this side-reaction could be fully prevented by starting the aza-Michael addition reaction at −20°C. Overall, these NMR measurements highlight the potential of *trans*-trimethyl aconitate as aza-Michael acceptor for green transformations of amines, as the aza-Michael addition reaction occurs instantaneously with quantitative conversion, at low temperatures in methanol as green solvent. In other words, we consider the aza-Michael addition with aconitic acid and primary amines a click-reaction, providing a promising route to transform e.g., primary amines or pendant amines, as for example present in chitosan, into trimethylcarboxylate or pyrrolidone based dimethylcarboxylates.

Previous work reported the cascade cyclization reaction to form the *N*-substituted pyrrolidone rings in moderate to high yields after prolonged stirring at ambient conditions (Singh et al., 2016). However, both LC-MS and ^1^H-NMR analysis show the presence of significant amounts of the ring-opened intermediate, even after stirring for 72 h at room temperature (details in ESI). Instead, the reaction mixture was heated to 125°C under reduced pressure for 2 h (to remove the condensate), which favors the cascade cyclization reaction into the desired pyrrolidone products (LC-MS, details in ESI). The expected products have been isolated via column chromatography as is confirmed by 2D-NMR analysis provided in ESI. Even though the purification via column chromatography is not considered green due to the high solvent usage, these findings clearly demonstrate the ease and potential of *trans*-trimethyl aconitate in the aza-Michael cascade reaction.

**Figure 2:**
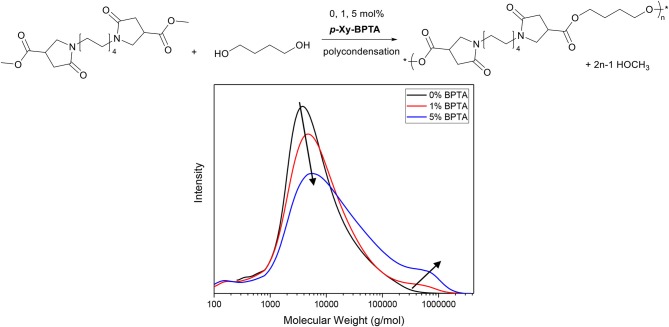
Top, Schematic overview of the melt-polycondensation between C_8_-BPDA-dm and 1,4-butanediol, with 0, 1, and 5 mol% of p-Xy-BPTA as cross-linker. Bottom, GPC traces of the soluble fractions of synthesized polymers with 0, 1, and 5 mol% of p-Xy-BPTA. Note, arrows added to guide the eye. GPC experiments were conducted with 1,1,1,3,3,3-hexafluoro-2-propanol as solvent, whereas the molecular weights were referenced against PMMA standards.

Furthermore, in contrast to aconitic acid itself, the isolated *para*-xylylene-*bis*-(pyrrolidone methyldicarboxylate) (***p*-Xy-BPTA**, Scheme 11) exhibits excellent thermal stability (no weight loss was observed during a 1 h isotherm at 200°C in thermogravimetric analysis, ESI) which allowed for its use as cross-linker in melt-polycondensation reactions of amorphous BPDA-based polyesters reported in our recent work (Noordzij et al., 2019). As is depicted in the GPC traces in Figure 2, the introduction of 1 or 5 mol% of ***p*-Xy-BPTA** to the monomer mixture containing C_8_-BPDA-dm and 1,4-butanediol (Figure 2, top), resulted in rapid branching and cross-linking (details on experimental conditions provided in Supporting Information). Note, though the GPC traces reflect only the soluble fraction of the synthesized polymers, they clearly indicate a broadening of dispersity by the increase in high molecular weight fractions (Figure 2, bottom), as is typical for branched and cross-linked polymers. Overall, these findings demonstrate that the aza-Michael cascade reaction can readily be employed to generate multi-functional methylcarboxylates with the thermal stability required for melt-polycondensation reactions. In particular, when using renewable amines, we consider this approach promising for the generation of fully renewable methylcarboxylate based cross-linkers for the use in thermosets or coatings.

## Future Potentials

### aza-Michael Addition on Muconic Acid

**Scheme 12:**
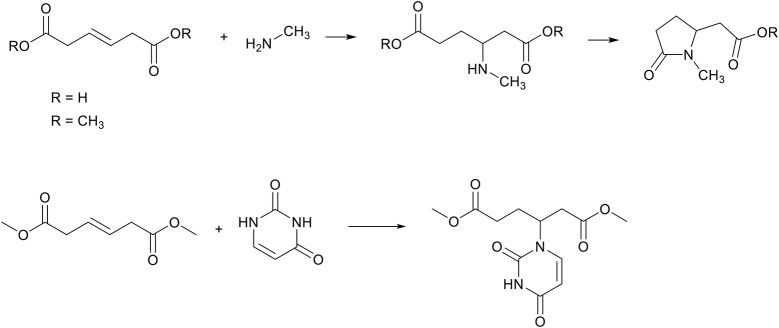
Top, the aza-Michael addition reaction between trans-β-hydromuconic acid and a primary amine, followed by a cyclization step to obtain *N*-methyl-2-pyrrolidone-ethanoic acid. Bottom, the aza-Michael addition reaction between *trans*-β-hydromuconic acid and a secondary amine, leading to the aza-Michael adduct.

The last unsaturated biobased di-ester discussed in this work is *trans*-β-hydromuconic acid, a derivative from muconic acid, both of which can be polymerized into biobased unsaturated co-polyesters (Rorrer et al., 2016; Yu et al., 2019). To the best of our knowledge there is no literature precedent on the aza-Michael addition on muconic acid itself, which is likely caused by the conjugation of the double bonds which has a stabilizing effect, and thereby negates the electron negative character of the unsaturated bonds in the β-position of the EWGs. The conjugation can be negated by partial hydrogenation and isomerization of the double bond to generate β-hydromuconic acid. However, the unsaturation of β-hydromuconic acid is not in the β-position, but in the γ-position relative to the EWGs, and is thereby less electron negative than in the previously described unsaturated esters. Nevertheless, several references describe the aza-Michael addition between primary and secondary amines and β-hydromuconic acid (Evans et al., 1950; Lavagnino and Ryan, 1983), and dimethyl β-hydromuconate (Wu et al., 1961). Interestingly, here also the cascade cyclization step to form the five-membered pyrrolidone ring is reported (Scheme 12, top). Furthermore, one example is known where *trans*-β-hydromuconic acid is reacted with the secondary amine group of several nucleosides, successfully forming the aza-Michael adduct in moderate yields in acetonitrile and in the presence of a base catalyst (Scheme 12, bottom) (Perbost et al., 1992).

**Scheme 13:**
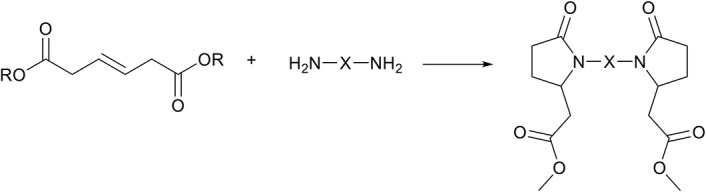
The potential reaction between *trans*-β-hydromuconic acid and di-amines leading to bis-pyrrolidone di-ethanoic acids, a potential structural analog to itaconic acid based BPDA's.

Apart from the increased interest of using *trans*-β-hydromuconic acid as precursor for adipic acid (Matthiesen et al., 2016a,b), or for the synthesis of unsaturated polyesters (Yu et al., 2019), little recent research interest is shown in this biobased molecule. However, considering the reported formation of the pyrrolidone ring, β-hydromuconic acid is a very interesting candidate to prepare (bis-)pyrrolidone structures similar to those prepared with itaconates (Scheme 6). For example, one can envision the reaction between β-hydromuconate and a di-amine to form the symmetrical bis-pyrrolidone di-ethanoic acids, structural analogs to the bis-pyrrolidones obtained from itaconic acid (Scheme 13). It is important to note that the reported conditions for the cascade aza-Michael addition-cyclization reaction for *trans*-β-hydromuconic acid (e.g., in ethanol at 200°C in an autoclave) are harsher compared to e.g., itaconates and aconitates. Such harsh reaction conditions are likely required due to the lower expected electronegativity of the unsaturation in β-hydromuconic acid, but this remains a topic for further investigation. Nevertheless, we consider β-hydromuconic acid a promising monomer for use in the aza-Michael cascade reaction and expect to see more of this reaction and its products in the future.

## Conclusions

In this work, we provided an overview of the aza-Michael addition reaction between amines with several renewable unsaturated dicarboxylic acids (and esters) including itaconic acid, fumaric acid, maleic acid, aconitic acid, and β-hydromuconic acid. When these monomers bear an ester group in the γ-position relative to the unsaturation, they prove susceptible to undergo a cascade aza-Michael addition-cyclization reaction to generate a stable five-membered *N*-functionalized pyrrolidone ring, while retaining one carboxylic acid or ester functionality. By careful selection of the amine as Michael donor, *N*-functional pyrrolidones with a wide range of functional groups can be prepared, which in turn can be used as monomers in melt-polycondensation reactions, allowing for the generation of a wide range of polyesters, poly(ester-amide)s, and polyamides with tunable physio-chemical properties. The state-of-the-art on the aza-Michael cascade reaction is extended in this work with new findings on trimethyl *trans*-aconitate. The aza-Michael addition with primary mono or di-amines can be considered a “click-reaction” that follows the principles of green chemistry: the reaction proceeds quantitatively at room temperature, requires no catalyst, and can be performed in methanol as solvent. Though the ring-closing cascade reaction requires elevated temperatures to reach full conversion, this route transforms the otherwise thermally unstable trimethyl *trans*-aconitate into thermally stable *bis*-pyrrolidone monomer with tetra-methylcarboxylate functionality. Lastly, the potential of this monomer in polycondensation reactions is demonstrated by its successful co-polymerization into renewable, branched, and cross-linked polyesters.

## Author Contributions

GN conducted experiments and analyzed the data. GN and CW wrote the paper.

### Conflict of Interest

The authors declare that the research was conducted in the absence of any commercial or financial relationships that could be construed as a potential conflict of interest.
