# Supplementary material for: Cascade aza-Michael Addition-Cyclizations; Toward Renewable and Multifunctional Carboxylic Acids for Melt-Polycondensation
Source: Front Chem. 2019 Nov 14;7:729. doi: 10.3389/fchem.2019.00729 (PMC6868100; doi:10.3389/fchem.2019.00729)
Supplement: Supplementary file 1 [file Data_Sheet_1.PDF]

Supplementary Material

1 Materials and Methods

1.1 Materials

1,4-Butanediol, benzylamine, and Sn(II)2-ethylhexanoate were purchased from Sigma Aldrich. *Trans*-trimethyl aconitate and *p*-xylylene-diamine were purchased from TCI chemicals. Standard laboratory solvents were obtained from BioSolve. Deuterated solvents were obtained from Buchem BV (Netherlands). C_8-BPDA-dm was synthesized from dimethyl itaconate and 1,8-diamino octane according to previously published synthesis methods.[1] The purchased compounds were used directly without further purification, unless otherwise specified.

1.2 Analysis methods

A Grace Reveleris X2 automatic flash column was used for the purification of products. ^{1}H-NMR and ^{13}C-NMR spectra were recorded with a Bruker Ultrashield 300 spectrometer (300 MHz magnetic field). Liquid Chromatography – Mass Spectrometry (LC-MS) analysis was performed on a Shimadzu Nexera 2 UHPLC system equipped with a Shimadzu LC-30AD pump, a SPD-M30A photodiode array detector and LCMS-2020 single quadrupole detector. The system was run on MilliQ water and LC-MS grade acetonitrile both modified with 0.1% formic acid. The following column was used on a Waters XSelect CSH C18 (3.0 mm x 75 mm with a particle size of 3.5 µm) operating at 30 °C. The method was setup with the following gradient: 5% acetonitrile in water for 2 min, an increase to 95% acetonitrile over 6 min, 1 min at 95% followed by flushing of the column back to 5% acetonitrile. All samples were prepared by dissolving 1-3 mg/ml of material in methanol or acetonitrile and filtering over 0.2 µm PFTE filter. NMR-samples were prepared by dissolving ca. 10 mg of sample in 0.5 mL deuterated solvent, including dimethyl sulfoxide (DMSO-d_6), deuterated chloroform ($CDCl_3$), and deuterated trifluoroacetic acid (*d*-TFA). All spectra were referenced against tetramethylsilane (TMS), or residual solvent peak from the deuterated solvent. Molecular weight (M_n, M_w) and dispersity (Đ) of the polymers were calculated after gel permeation chromatography (GPC). HFIP-GPC was measured on a PSS SECcurity GPC system using Agilent 1260 Infinity instrument technology. The GPC was equipped with a PFG Combination pre-column and two PFG Combination micro-columns. Distilled HFIP containing 0.019 % sodium trifluoroacetate was used as mobile phase at 40 °C, with a 0.3 mL/min flow rate. $CHCl_3$-GPC was measured on a Prominence-I LC-2030 equipped with a Shodex GPC KF-805L column. Analytical grade $CHCl_3$ was used as mobile phase at 40 °C, with a flowrate of 1 ml/min. GPC samples were prepared by dissolving ca. 5 mg of polymer in 1.5 mL of solvent overnight under constant shaking, the samples were filtered over a 0.2 µm PTFE syringe filter prior to injection. Thermal stability of compounds were performed via thermogravimetric analysis (TGA) using a TA Instruments Q500. Experiments were performed under a nitrogen atmosphere with a heating rate of 10 °C/min.

---

[1] G. J. Noordzij, Y. J. G. van den Boomen, C. Gilbert, D. J. P. van Elk, M. Roy, C. H. R. M. Wilsens and S. Rastogi, *Polym. Chem.*, , DOI:10.1039/C9PY00463G.

1.3 Synthesis methods

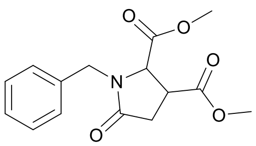

Synthesis of *N*-benzyl-pyrrolidone-2,3-dicarboxylate

At -20 ºC, a solution of benzylamine (1.018 g, 9.5 mmol) in 2.5 ml methanol was added dropwise to a stirred solution of *trans*-trimethyl-aconitate (2.162 g, 10 mmol) in 2.5 ml methanol. The solution turned yellow immediately. The solution was stirred overnight, whilst warming up to room temperature, and the colour changed to dark brown. Subsequently the solution was heated to 125 ºC for 2 hours, and finally one hour of vacuum was applied at 125 ºC to yield a viscous brown oil. The product was purified via column chromatography (ethyl acetate : heptane) with a gradient from 1:2 to 1:1. The product is isolated as orange oil, which crystallized over time (RF 0.2, 1.81 g, 65% yield). ^{1}H-NMR ($CDCl_3$, 300 MHz): δ 7.41-7.17 (m, 5H), 5.05-4.91 (m, 0.98H), 4.33-4.17 (m, 1H), 4.15-3.93 (m, 1.03H), 3.74-3.62 (m, 5.91H), 3.47-3.24 (m, 1.04H), 3.11-2.58 (m, 2.02H). ^{13}C-NMR ($CDCl_3$, 300 MHz): δ 173.0, 172.6, 171.8, 170.7, 170.4, 169.9, 135.3, 135.2, 128.8, 128.7, 128.6, 128.4, 128.0, 127.9, 61.0, 60.5, 52.8, 52.5, 45.7, 40.8, 40.2, 33.0, 32.5.

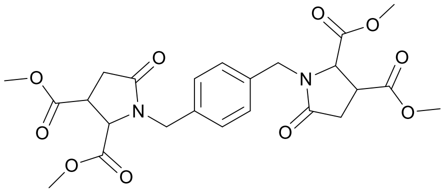

Synthesis of *N,N'*-*para*-xylylene-*bis*-pyrrolidone-2,3-dicarboxylate (*p*-Xy-BPTA)

At -20 ºC, a solution of *para*-xylylenediamine (0.647 g, 4.75 mmol) in 2.5 ml methanol was added dropwise to a stirred solution of *trans*-trimethyl-aconitate (2.162 g, 10 mmol) in 2.5 ml methanol. The solution turned yellow immediately. The solution was stirred overnight, whilst warming up to room temperature, and the colour changed to dark brown. Subsequently the solution was heated to 125 ºC for 2 hours, and finally 2 hours of vacuum was applied at 125 ºC to yield a viscous brown oil. The product was purified via column chromatography (methanol : chlorofrom) with a gradient from 2% to 10% methanol. The product is isolated as green transparent solid, (R_F 0.15, 1.33 g, 56% yield). ^{1}H-NMR ($CDCl_3$, 300 MHz): δ 7.15-7.07 (m, 3.89H), 4.98-4.83 (m, 2H), 4.25-4.08 (m, 2.02H), 4.04-3.81 (m, 2.11H), 3.67-3.53 (m, 12.01H), 3.41-3.17 (m, 2.13H), 3.01-2.51 (m, 4.14H). ^{13}C-NMR ($CDCl_3$, 300 MHz): δ 172.6, 171.8, 170.6, 169.8, 134.9, 128.9, 128.7, 128.6, 61.0, 60.5, 52.8, 52.5, 45.3, 40.7, 40.2, 33.0, 32.5.

Polymerization of C_8-BPDA-dm, 1,4-butanediol, and *p*-Xy-BPTA

Polyesters of C_8-BPDA-dm and 1,4-butanediol with varying amounts of *p*-Xy-BPTA have been prepared. Upon addition of BPTA, a portion of C8-BPDA-dm was left out to ensure a stable acid/alcohol ratio. The exact amounts weighed in can be found in Table S1. Exemplified is the polymerization with 5 mol% BPTA: C_8-BPDA-dm (1.42 g, 3.6 mmol), 1,4-butanediol (0.73 g, 8.1 mmol), *p*-Xy-BPTA (0.052 g, 0.1 mmol) were loaded in a 100 mL 3-neck round-bottom flask, equipped with a N_2/vacuum inlet, vigreux column, and a heavy duty mechanical stirrer. The reaction mixture was purged 3 times with a vacuum/N_2 cycle. The reaction mixture was slowly heated under light N_2 flow to 120 °C, after which the catalyst Sn(II)2-ethylhexanoate (16.7 mg, 0.041 mmol, in 1 ml anhydrous DMF) was added. For the oligomerization step, the reaction mixture was gradually heated to 180 °C where it was kept isothermal for 2 hours to allow for the generation and distillation of the condensate and DMF. Next, vacuum was applied for 4 – 8 hours to allow build-up of molecular weight.

The final polymers were obtained as a light orange sticky viscous solids and were used without further purification.

Table S1. Overview of added monomer ratio in the polymerization of C_8-BPDA-dm, 1,4-butanediol, and p-Xy-BPTA.

Mol % p-Xy-BPTA	**1,4-butanediol**		**C_8-BPDA-dm**		***p*-Xy-BPTA**	
	g	mol	g	mol	g	mol
No cross-linker	0.73	8.14	1.5	3.78	-	-
1 mol%	0.73	8.14	1.48	3.75	0.010	0.02
5 mol%	0.73	8.14	1.42	3.59	0.052	0.10

2 ELSD analysis of p-xylylene-BPTA monomer synthesis

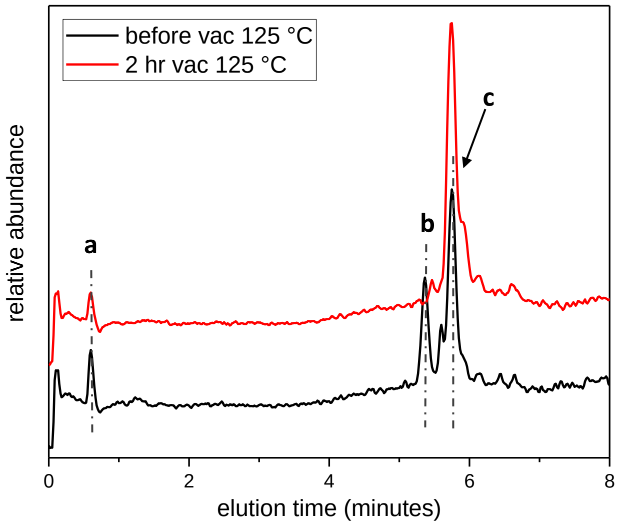

Figure S1. ELSD traces of the synthesis of p-Xy-BPTA. Bottom (black): 2hr @ 125 °C, top (red): 2hr @ 125 °C under vacuum. A: p-xylylene-diamine reacted once with trimethyl-aconitate. B: intermediate before cyclization reaction. C: product. It can be observed how peak B has decreased significantly after the vacuumstep, and nearly only product peak C is present. This resulting reaction mixture after the vacuumstep was subsequently purified via column chromatography to isolate the product p-Xy-BPTA.

3 2D-NMR analysis of monomers

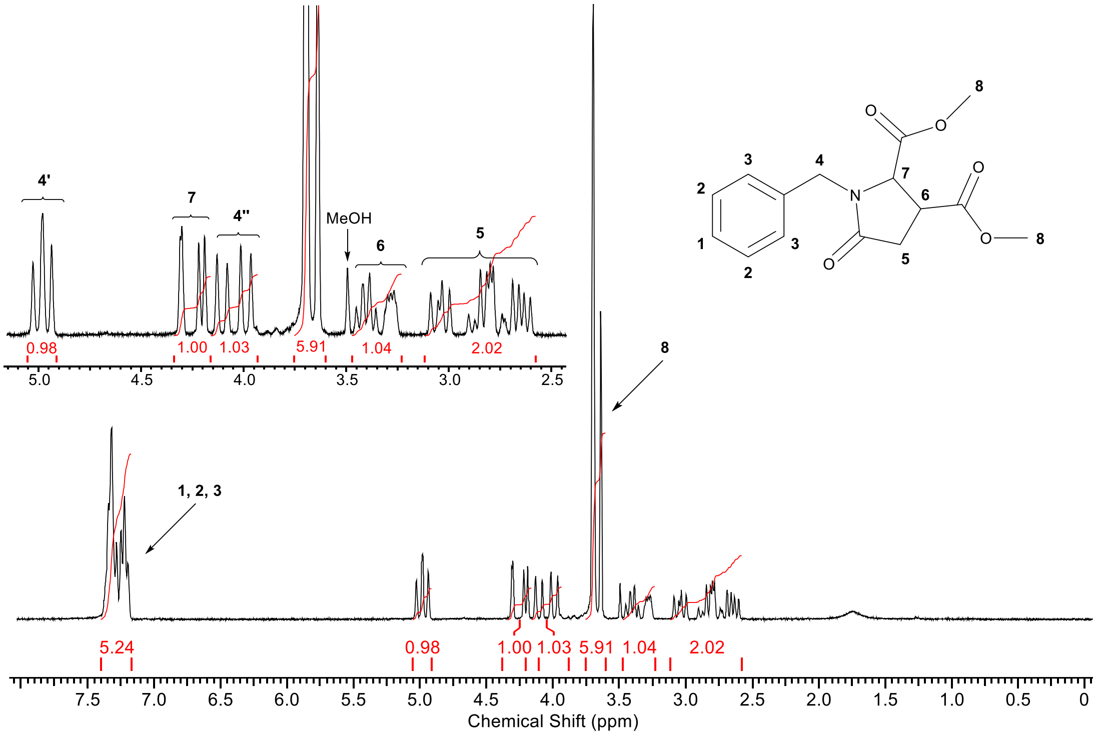

Figure S2. ^{1}H-NMR spectrum of N-benzyl-2,3-pyrrolidone-dicarboxylate.

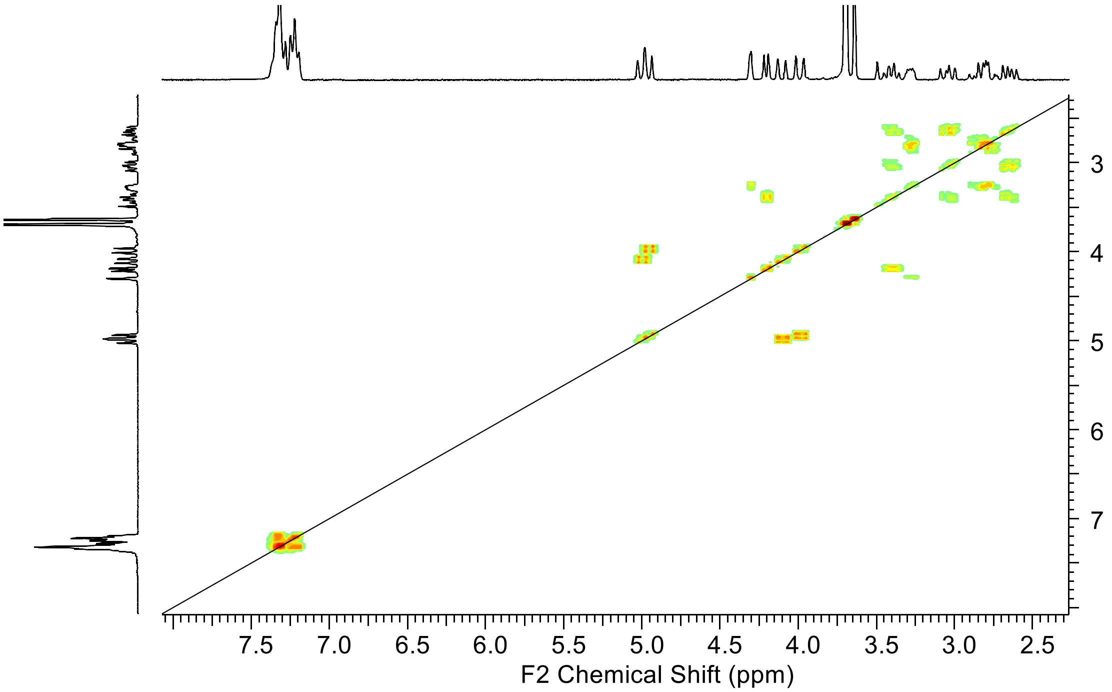

Figure S3. COSY (^{1}H-^{1}H) NMR spectrum of N-benzyl-2,3-pyrrolidone-dicarboxylate.

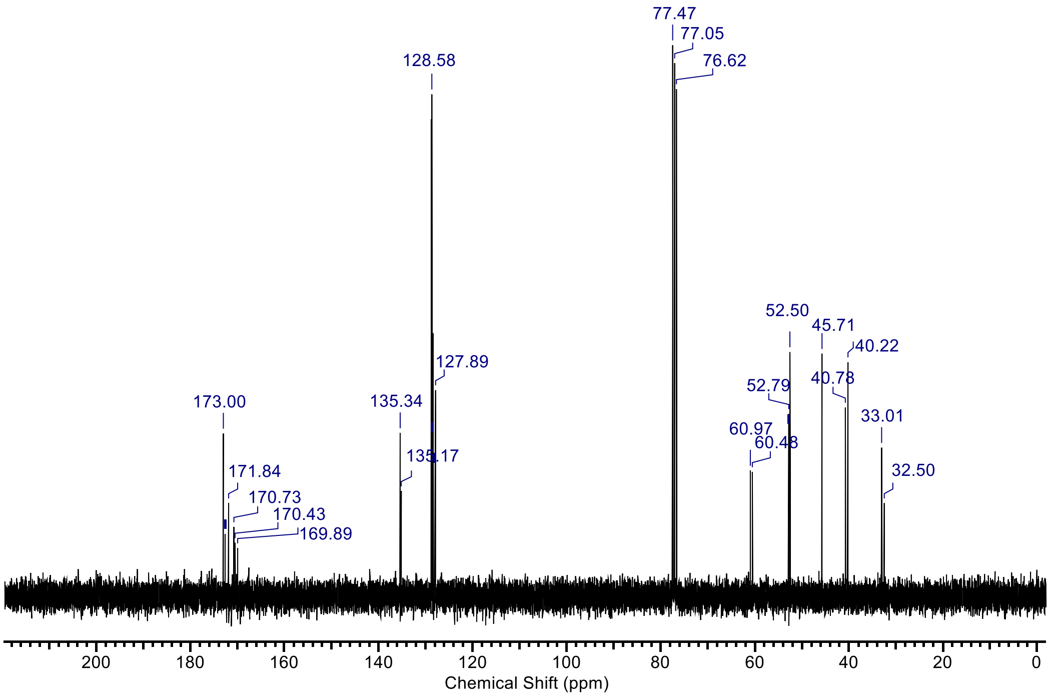

Figure S4. ^{13}C-NMR spectrum of N-benzyl-2,3-pyrrolidone-dicarboxylate.

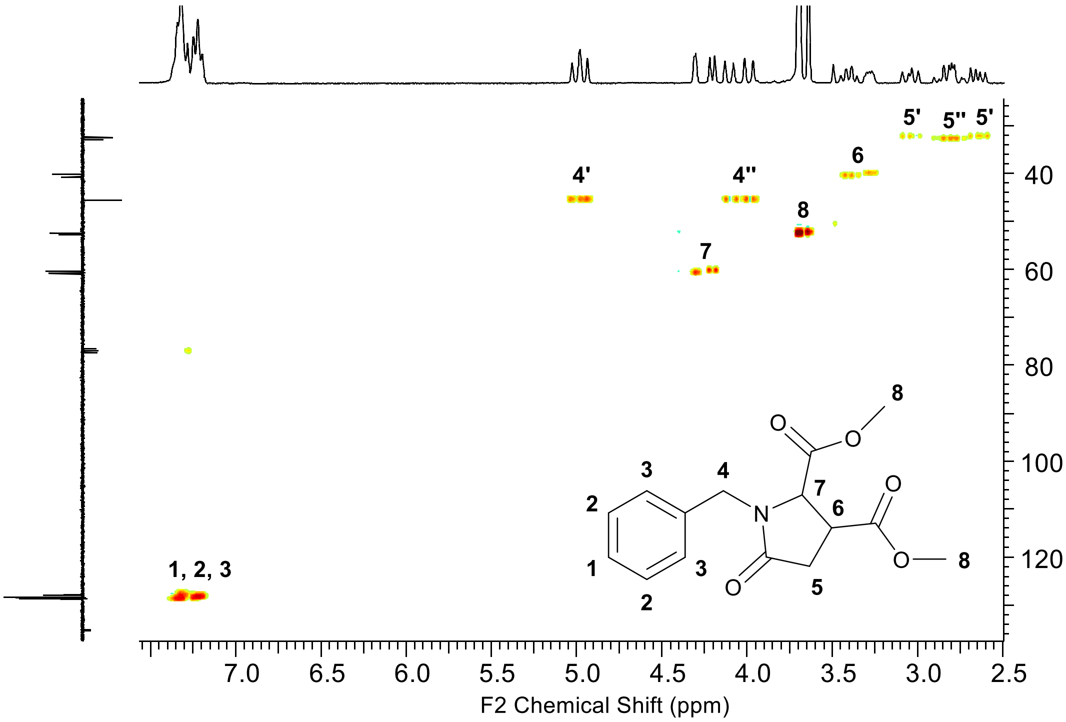

Figure S5. HSQC (^{1}H-^{13}C-APT) NMR spectrum of N-benzyl-2,3-pyrrolidone-dicarboxylate.

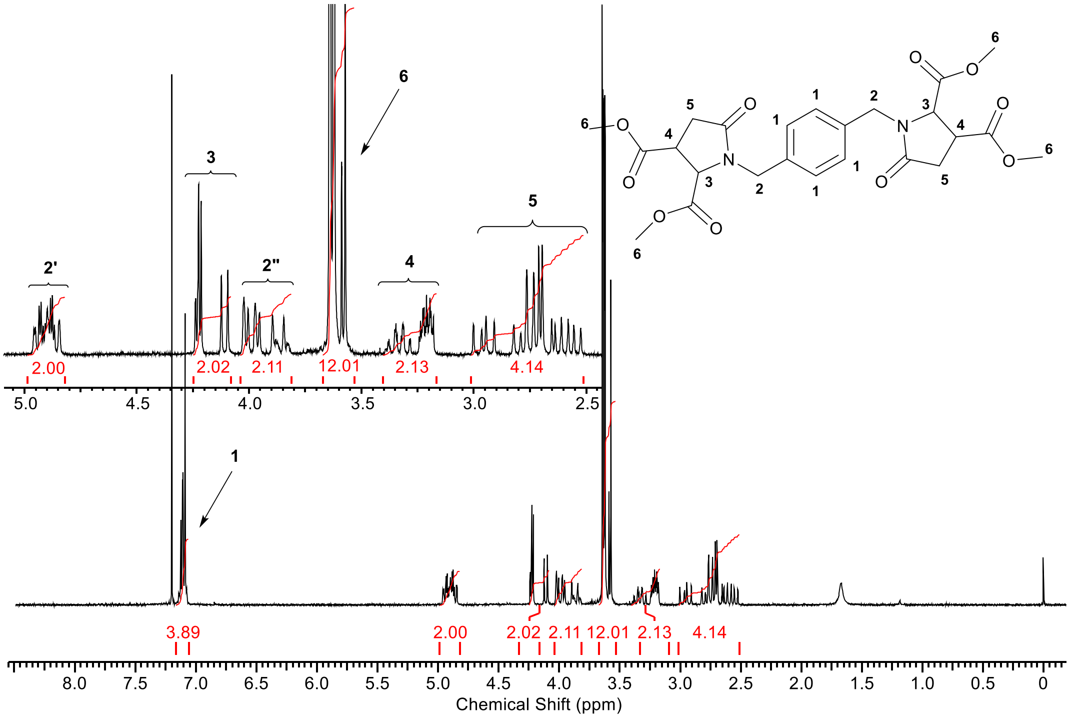

Figure S6. ^{1}H-NMR spectrum of N,N'-para-xylylene-bis-2,3-pyrrolidone-carboxylate.

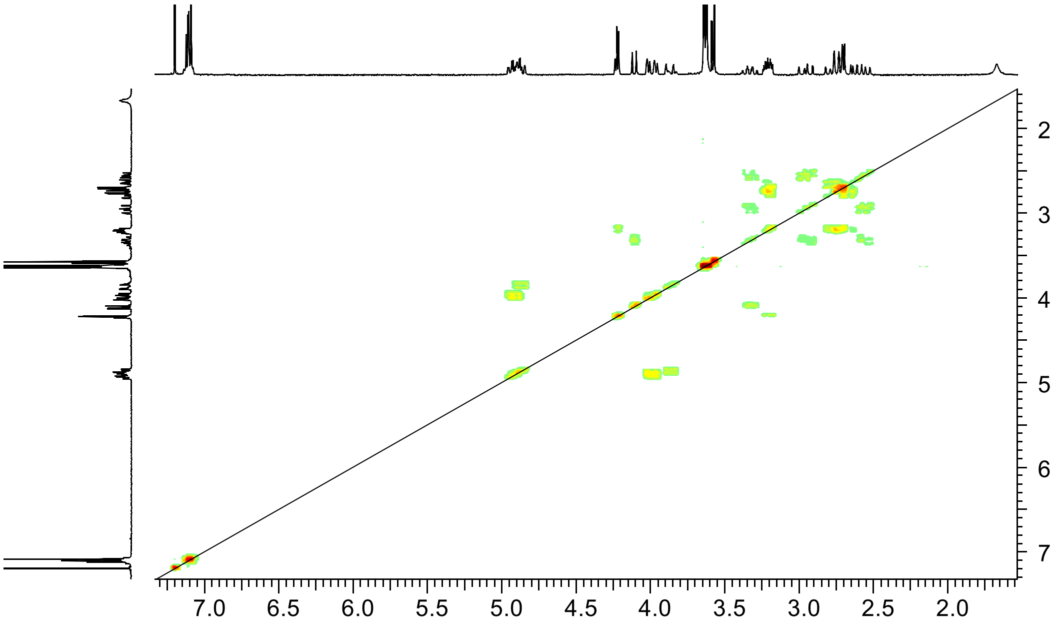

Figure S7. COSY (^{1}H-^{1}H) NMR spectrum of N,N'-para-xylylene-bis-2,3-pyrrolidone-carboxylate.

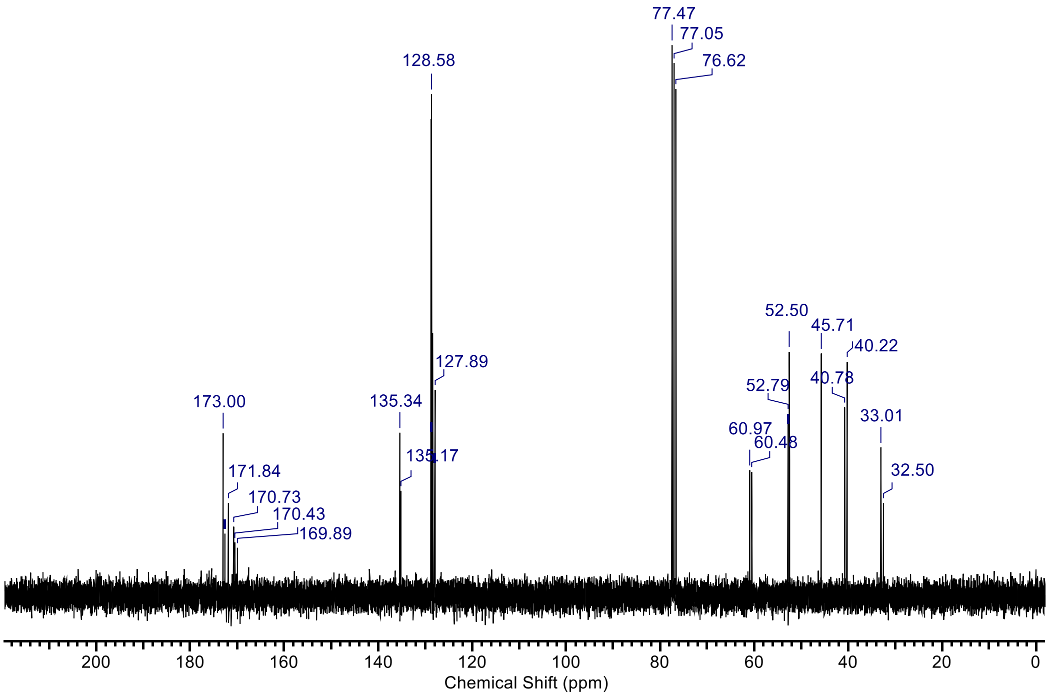

Figure S8. ^{13}C-NMR spectrum of N,N'-para-xylylene-bis-2,3-pyrrolidone-carboxylate.

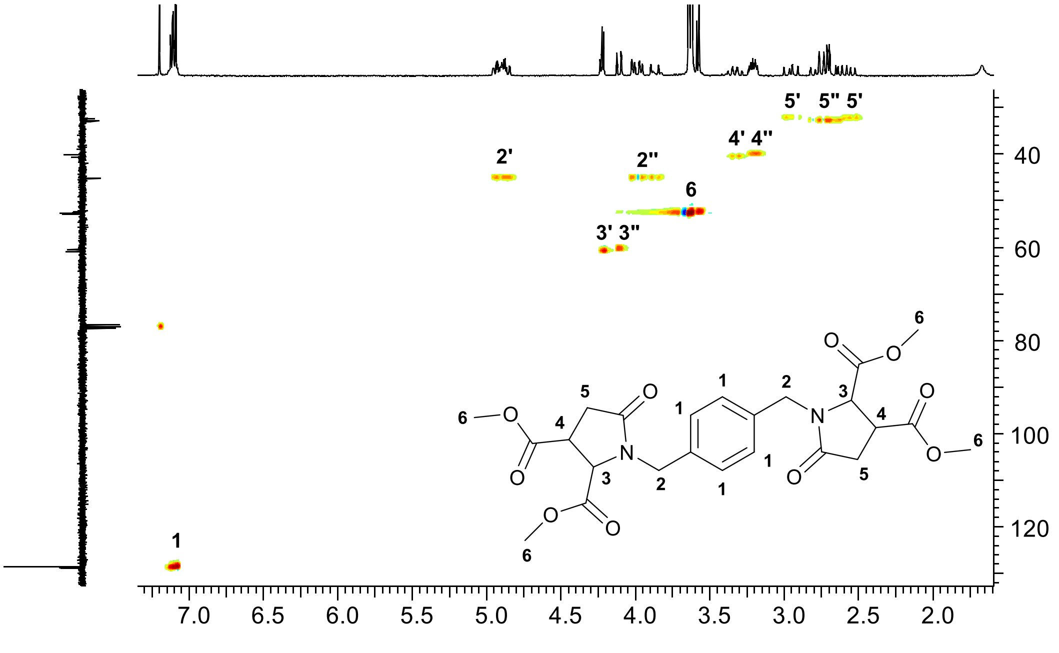

Figure S9. HSQC (^{1}H-^{13}C-APT) NMR spectrum of N,N'-para-xylylene-bis-2,3-pyrrolidone-carboxylate.

4 TGA analysis of *para*-Xylylene-BPTA

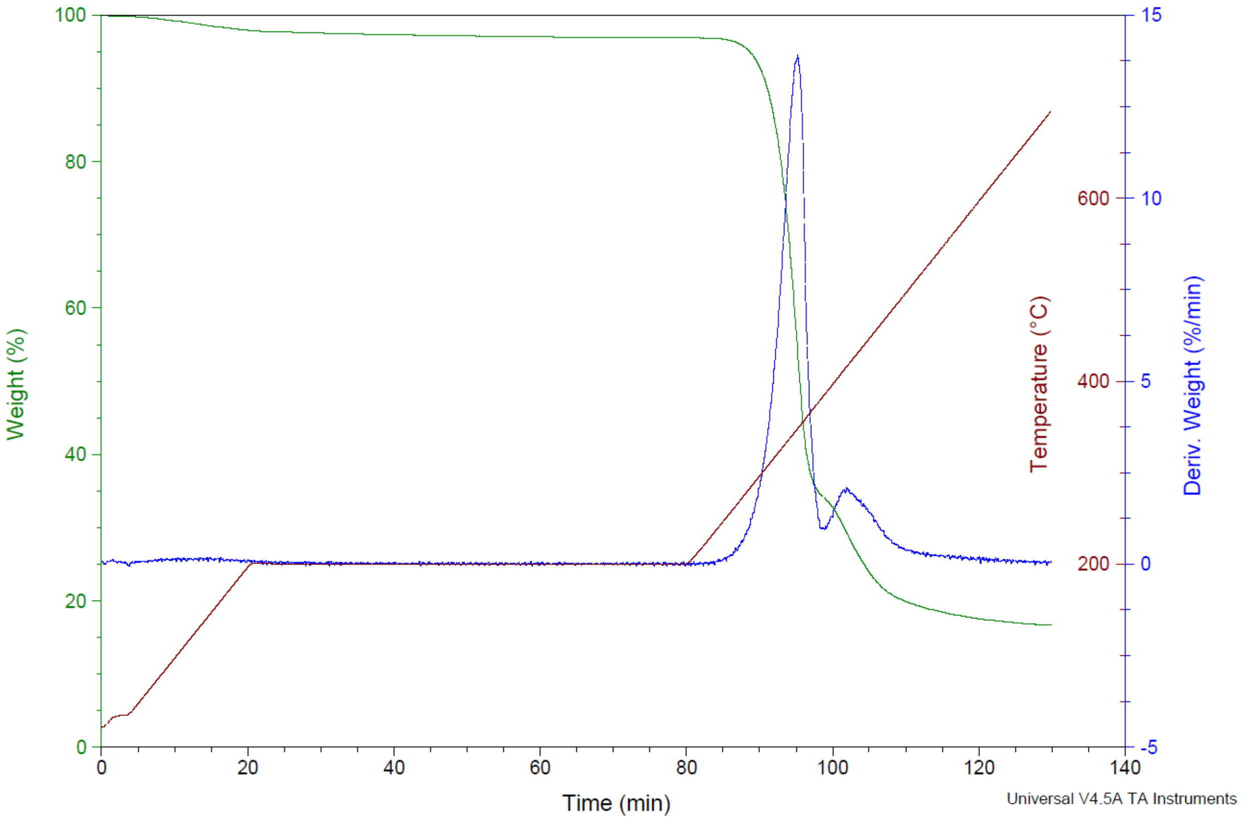

Figure S10. TGA analysis of p-Xy-BPTA monomer, with a one hour isothermal step at 200 °C showing no weight loss, indicating p-Xy-BPTA is stable at this temperatures. Program: 10 °C/min to 200 °C, 60 minutes isothermal at 200 °C, 10 °C/min to 700 °C.

5 Reaction scheme of polyimide formation from di-maleimides

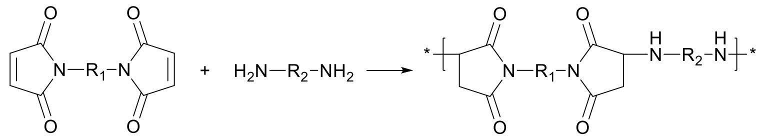

Scheme S1. Aza-Michael addition of a di-maleimide with a diamine generating a polyimide.
